# Comparison of Invasive Blood Pressure Measurements from the Caudal Ventral Artery and the Femoral Artery in Male Adult SD and Wistar Rats

**DOI:** 10.1371/journal.pone.0060625

**Published:** 2013-04-05

**Authors:** Ying Wang, Yushuang Cong, Jun Li, Xueting Li, Bing Li, Sihua Qi

**Affiliations:** 1 Department of Anesthesiology, The Fourth Affiliated Hospital, Harbin Medical University, Harbin, China; 2 Department of Nephrology, The Second Affiliated Hospital, Harbin Medical University, Harbin, China; The University of Tennessee Health Science Center, United States of America

## Abstract

**Background and Purpose:**

Studies have suggested that the caudal ventral artery is a potential site for continuous arterial blood pressure monitoring in rats. However, the agreement of mean arterial pressure values between the femoral artery and the caudal ventral artery has not been investigated. This study was performed to identify whether the caudal ventral artery could be safely used for continuous blood pressure monitoring as an alternative site to the femoral artery.

**Methods:**

Rats were randomized into four groups: Sprague Dawley rats under normothermia; Wistar rats under normothermia; Sprague Dawley rats under hypothermia; Wistar rats under hypothermia. Each rat underwent simultaneous monitoring of blood pressure using femoral artery and caudal ventral artery catheterization during a stable hemodynamic state and three periods of acute severe hemodynamic changes. The effects of rat strain, rectal temperature, experimental time course and hemodynamic factors on pressure gradients, the concordance of mean arterial pressure values between the femoral artery and the caudal ventral artery, and the rates of distal ischemia after surgery were determined.

**Results:**

There was a significant difference in the rate of distal ischemia between femoral and caudal ventral arteries after catheterization (25% *vs* 5%, *P*<0.05). The overall mean gradient and the mean gradient under a steady hemodynamic state were 4.9±3.7 mm Hg and 5.5±2.5 mm Hg, respectively. The limits of agreement (bias±1.96 SD) were (−2.5 mm Hg, 12.3 mm Hg) and (-0.5 mm Hg, 10.5 mm Hg), respectively. Although the concordance decreased during the first 30 sec of each period of severe hemodynamic changes, the degree of agreement was acceptable regardless of the effects of rat strain and rectal temperature.

**Conclusions:**

Based on the degree of agreement and the safety of catheterization, the caudal ventral artery may be a preferred site for continuous arterial blood pressure monitoring without acute severe hemodynamic changes.

## Introduction

Acute and chronic femoral or common carotid arterial blood pressure (BP) monitoring with arterial cannulation is generally preferred in animal experiments because it is fundamental for quality control and for BP reporting in a comprehensive fashion [1]. Choosing a suitable site is largely dependent on the experimental objective [1], [2]. However, the accuracy of measurement and the complication rate is a concern. The femoral artery (FA) is the most commonly used artery in rats because it most accurately reflects perfusion of the vital organs without leading to brain ischemia. BP criteria in some animal models of disease, such as hemorrhagic shock or transient forebrain ischemia, have been established according to the data obtained from the FA. However, the invasive nature of the procedure and the high rate of limb ischemia (approximately 10%∼30%) limit its application in some long-term experiments, particularly when accompanied by neurological or behavioral examination. Therefore, it is necessary to find cannulation sites with a level of measurement accuracy as high as the FA that minimize distress and have a low rate of distal ischemia.

The caudal ventral artery of the tail (coccygeus medial artery, CVA), also called the mid-ventral artery, stems from the Pars abdominal aorta and the median sacral artery. It is enclosed in a groove that is covered ventrally by a thick fascia, formed by the hemal processes or coccygeal muscles from the tail base to the end. The thickness of the arterial wall decreases only slightly from the base to the tip of the tail. Similarly, the luminal diameter of the CVA changes only slightly from the base to the middle segments [3], [4]. The CVA has prominent collateral circulation, and its caliber changes as it reaches the tail end [5]. Many studies investigating thermoregulation, microvascular surgery and anatomical structure have been performed by utilizing this vessel as an entire isolated vascular bed or as a small isolated dissected segment, [4]–[11]. Scientists have established and improved the chronic CVA cannulation technique under stress-free conditions in freely moving rats for up to 12 days [12]–[15]. However, only a few studies have focused on the *in vivo* function of the vessel, especially the vascular tone [15]–[17]. These studies implied that the CVA might be an alternative site to the FA, not only for repeatedly performing drug administration or blood sampling but also for continuous invasive arterial blood pressure monitoring.

However, the arterial pressure waveform undergoes characteristic morphological changes as it travels from central to peripheral sites. Central-peripheral arterial pressure gradients have received considerable attention in humans [18]–[21]. These differences may lead to inconsistent measurements of BP obtained from different arterial sites. In our preliminary experiment, we noticed that there is a small mean arterial pressure (MAP) gradient between the FA and the CVA under normal physiological conditions in male adult SD rats, although no tail ischemia was observed. Currently, the factors causing the pressure gradient are not completely known. Some important factors, such as blood volume, body temperature, arterial elasticity and heart rate, are the most crucial in the differences in blood pressure detected from the different arteries. If BP measurements from the CVA lead to overestimation or underestimation compared to the FA, improper dosing of medicine or fluid therapy may put the rats in danger. To the best of our knowledge, no one has focused on the consistency of invasive BP measurements between the FA and CVA in rats. The variability of the pressure gradient between these two arteries during aberrant experimental conditions and the safety of CVA catheterization has not been documented. To confirm whether the CVA could be used as a measurement site in place of the FA for continuous invasive blood pressure monitoring, we evaluated the accuracy and the safety of invasive arterial blood pressure monitoring at the CVA, comparing measurements to the FA simultaneously by evaluating: 1) the rates of distal ischemia; 2) the effect of rat strain, rectal temperature, vascular tone and blood volume on the variability of the difference between CVA and FA; and 3) the overall difference, the limits of agreement and the concordance of MAP measurements between both arteries during steady hemodynamic state and acute severe hemodynamic changes.

## Materials and Methods

### Animal Care

All procedures were performed according to NIH guidelines and approved by the Committee on the Guidelines for Animal Experiments of Harbin Medical University. All rats (Vital River Laboratory Animal Technology Co. Ltd., Beijing, China) were healthy and housed in a climate-controlled room (temperature between 25°C and 28°C and a relative humidity of 60±5%) with food and water *ad libitum* and a 12 h light-dark cycle.

The animals were maintained on surgical anesthesia throughout the entire measurement study and were euthanized at the end of the study using an overdose of anesthesia.

### Study Design

Two experiments were designed to avoid confusion and reduce the number of animals. Experiment 1 was conducted with a two-factor, two-level factorial experiment design to evaluate BP agreement between the CVA and the FA. The effects of two different rat strains and two body temperatures on the concordance of measurements were investigated. Forty healthy adult male rats weighing 220–250 g were divided into four groups randomly (n = 10): Sprague Dawley (SD) rats under normothermia, 37°C, (SN); Wistar rats under normothermia, 37°C, (WN); SD rats under hypothermia, 32°C, (SH); and Wistar rats under hypothermia, 32°C, (WH).

Experiment 2 was conducted to validate the safety of intra-arterial catheterization 24 h after surgery. Because the FA and the CVA both stem from branches of the Pars abdominal aorta, the rate of distal ischemia at each site was investigated to eliminate mutual interference between the FA and the CVA. Twenty SD rats and twenty Wistar rats, weighing 220–250 g, were randomized into two groups (n = 20): Group A and B. Hind-limb ischemia after FA catheterization in group A and tail ischemia after CVA catheterization in group B were measured individually.

### Surgical Preparation

In both experiments, anesthesia was maintained with 70% N_2_O/30%O_2_ in spontaneously breathing rats during the entire experimental period. Briefly, in experiment 1, rats were initially anesthetized using chloral hydrate (300 mg/kg intra-peritoneal injection) and local infiltration anesthetic (0.2% Bupivacaine) at approximately 10∶00 AM. Then, the bilateral FA, the left femoral vein (FV) and the tail CVA were exposed, and indwelling 24 G catheters (Becton, Dickinson and Company, USA) were inserted and pushed forward 2 cm. All vessels for cannulation were ligated distally and fixed with prepared ligatures, taking caution not to twist the vessels. The left FA was used for bloodletting. To measure blood pressure, the right FA and the CVA were individually connected to a *Datex* AS3 physiological monitoring instrument (Communications Specialties, Inc., USA) through heparin-filled pressure transducers (25 IU/ml). Both pressure transducers were placed at the same level (heart) on a plastic support. The left FV was used for fluid and medicine infusion. A 1.0 cm incision was made on the ventral side of the tail, beginning approximately 1.5 cm from its base, at the same monitoring site as for a tail cuff. In our preliminary experiment, the same stable and clear waveforms were obtained from both arteries. A digital thermistor probe (Multi-thermistor Meter D321; Technol Seven, Yoko-hama, Japan) was used for monitoring the rectal temperature. The rectal temperature was controlled by a water blanket placed under the body, and the targeted rectal temperature was 37°C for normothermia and 32°C for hypothermia. After surgical procedures, rats were included in the study if the blood gas parameters (90–140 mm Hg PO_2_, 35–45 mm Hg PCO_2_, pH 7.35–7.45) and the levels of blood glucose (4–10 mmol/L) were measured in the published acceptable levels ranges.

### Sequential Treatment and Simultaneous Blood Pressure Recording

After being stabilized for 5 min, each rat included in the study was exposed to a series of rapid sequential treatments to mimic acute changes in blood volume or vessel tone that often occur in animal experiments. Each rat experienced acute induced hemorrhagic shock (MAP: 40±5 mm Hg from the FA) by rapidly withdrawing blood from the left FA in 10 sec. The extracted blood was heparinized and mixed with saline in a ratio of 2∶3 as a fluid mixture and kept for future use. After two minutes, 0.2 ml of saline containing 4 µg phenylephrine was administered [16]. In the preliminary study, we also confirmed that a dosage of 4 µg phenylephrine does not have peak drug effects on MAP values larger than 150 mm Hg. Five minutes after phenylephrine administration, a volume of the fluid mixture equal to that extracted earlier from the left FA was transfused into the left femoral vein at a constant speed within 120 sec. In the preliminary study, we also verified that the BP changes become relatively stable after 60 sec. We recorded observations for a period of 120 sec under each experimental situation. The values of systolic blood pressure (SBP), diastolic blood pressure (DBP) and MAP at both sites were continuously monitored and recorded every 15 seconds by a video camera. After surgery, the wounds were disinfected, closed and covered with a small piece of gauze, and the animals were observed for 24 h.

**Figure 1:**
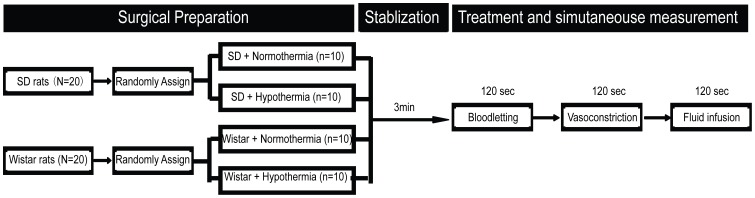
The surgical procedures and simultaneous measurements of BP.

To determine the agreement between the CVA and the FA blood pressure measurements, the SBP, DBP and MAP were continuously monitored at both sites and were simultaneously recorded with a video camera. The data were obtained from two different hemodynamic states. One was a relative steady hemodynamic state with normal blood volume. The other was the dynamic process following acute severe hemodynamic changes such as blood loss, vasoconstriction and fluid supplementation. During acute hemodynamic changes, the BP values at each site were recorded every 15 sec for 120 sec. Intra-measurement precision demonstrated the repeatability of measurements in each artery during intra-artery blood pressure monitoring. Measurements of the BP at the two arteries were each analyzed only once at each time point as described above. MAP was defined as DBP plus a third of the PP. That is, MAP = DBP+1/3(SBP-DBP) = DBP+1/3PP. Pulse pressure (PP) was defined as the difference between SBP and DBP. We focused on the analysis of interchangeability on MAP values because the tissue perfusion pressure is primarily dependent on MAP rather than SBP or DBP. The protocol for experiment 1 is shown in Fig. 1.

### Observation of Distal Ischemia

In experiment 2, the FA in group A and the CVA in group B were exposed, and catheters of the same size were inserted. The rates of distal ischemia 24 h after surgery were recorded. Distal ischemia was determined by the color and temperature of the skin and the Tarlov Score, as previously reported by Westvik et al. [22]. Rats with lower skin temperature, that could barely move their hind limbs or tails or that could not bear their own weight were regarded as rats with distal ischemia.

### Statistical Analysis

All data were analyzed using SAS 9.1 software (SAS Institute Inc., Cary, NC, USA) and the MedCalc software program (MedCalc®, Mariakerke, Belgium, http://www.medcalc.be/). Descriptive data were expressed as the mean ± standard deviation and were calculated for continuous data. Paired t-tests were performed to compare the values of SBP, DBP, MAP and PP between the FA and the CVA. ANOVA was performed to assess the effect of rat strain, rectal temperature, experimental time course and hemodynamic changes on MAP gradients. Bland–Altman analyses [23], [24] and concordance analysis were performed to compare the MAP values between the CVA and the FA. Individual absolute gradients of ≤10, 11–15 and >15 mm Hg were arbitrarily classified as good, acceptable and poor, respectively. The percentages for each gradient degree were calculated. The rates of hind limb ischemia or distal ischemia of the tail were analyzed using the Fisher exact test. Statistical significance was defined as *P*<0.05.

## Results

### Physiological Parameters

All rats were included in the study according to the inclusion criteria with optimal anesthetic techniques. Body weight, pH, PaCO_2_, PaO_2_ and glucose levels were similar among the four groups before sequential treatments (*P*>0.05, Table 1). The overall mean MAP from the FA in healthy male adult rats under optimal chloral hydrate and local infiltration anesthesia was 93.4±12.5 mm Hg (Wistar rats: 92.4±17.6 mm Hg, SD rats: 93.7±11.2 mm Hg). The baseline MAP values (T_0_) from the FA in each group were not out of the normal range, and no significant difference was found among these groups (*P*>0.05, Table 2).

**Table 1:** Physiological parameters of inclusion criteria.

	Groups (n = 10)			
Parameters	DN	DH	WN	WH
**Body weight** (g)	234±12	235±13	233±11	237±13
**PaO_2_** (mm Hg)	140±10	144±12	145±11	146±14
**PaCO_2_** (mm Hg)	38±3	39±3	37±2	38±3
**pH**	7.40±0.04	7.41±0.03	7.42±0.03	7.39±0.04
**Blood glucose** (mmol/L)	6.4±2.2	6.8±2.1	6.5±1.9	6.9±1.7

The presented values are the means ± SD. DN, SD rats under normothermia; DH, SD rats under hypothermia; WN, Wistar rats under normothermia; WH, Wistar rats under hypothermia.

**Table 2:** MAP, SBP, DBP and PP values at T_0_ (baseline) before bloodletting.

	MAP		SBP		DBP		PP	
Group	FA	CVA	FA	CVA	FA	CVA	FA	CVA
**DN**	88.8±13.1	84.4±13.5	112.7±11.4	95.9±12.9*	71.1±10.3	73.0±13.7	41.6±10.1	25.9±11.4*
**DH**	97.5±11.2	92.4±11.6	118.6±7.3	97.8±8.5*	79.7±6.3	80.3±6.4	38.9±7.8	17.3±5.1*
**WN**	96.5±10.7	89.8±10.9	114.2±8.9	97.2±11.6*	82.5±10.9	80.2±12.2	31.7±8.0	17.0±7.0*
**WH**	90.9±12.5	84.7±12.4	117.3±6.3	94.6±5.4*	82.0±6.0	80.6±5.3	35.3±8.4	16.0±6.7*

The presented values are the means ± SD. SBP, systolic blood pressure; DBP, diastolic blood pressure; MAP, mean arterial pressure; PP, pulse pressure; CVA, caudal ventral artery; FA, femoral artery; DN, SD rats under normothermia; DH, SD rats under hypothermia; WN, Wistar rats under normothermia; WH, Wistar rats under hypothermia.

* *P*<0.05, FA vs. CVA.

### Rates of Distal Ischemia

There was a significant difference in the rates of distal ischemia between group A (25%) and group B (0%) after FA and CVA catheterization, respectively (*P*<0.05).

### Analysis of the Associated Factors

Repeated measurement data analyses of variance failed to identify any statistically significant effect on MAP gradients from strain (*P*>0.05) or from normothermic or hypothermic rectal temperature (*P*>0.05) on MAP gradients. In contrast, acute hemodynamic changes had significant effects on MAP gradients (*P*<0.05, data not shown).

### Simultaneous Comparison of BP from the CVA and the FA

In total, 1080 pairs of each BP parameter were analyzed. The average baseline values (T_0_) of SBP, DBP, MAP and PP in each group are shown in Table 2. The mean value of SBP and PP obtained from the FA is significantly higher than those obtained from the CVA (*P*<0.05). Although both sites were comparable with regard to the values of MAP and DBP (*P*>0.05), an absolute systemic gradient in MAP values measured between the FA and the CVA was demonstrated.

The agreement analysis of the MAP values between the FA and CVA were divided in two portions using the Bland-Altman method. First, the analysis of the overall agreement of a total of 1080 pairs of MAP measurements and 40 pairs of MAP measurements at T_0_ (baseline) made in 40 rats between the FA and the CVA was performed (Table 3 and Fig. S1). The overall mean difference (bias, examined as gradients) of MAP values was 4.9 mm Hg, the precision (SD) was 3.7 mm Hg, and the percentage error was 4.9%. The limit of agreement (bias±1.96 SD) is (−2.4, 12.2 mm Hg) (Fig. S1A). Based on published literature, the bias and the precision did not exceed the a priori criteria of 15 mm Hg. Thus, we considered both sites to be interchangeable for MAP measurements regardless of acute hemodynamic changes.

**Table 3:** Bland-Altman parameters for concordance between MAP values measured at FA and CVA at all the time points.

Time Point	No. of Observations	Bias±SD	95% CI (mmHg)		*r*	Percent Error	Percent Exceeding (%)		
	(Pair)	(mmHg)	Upper	Lower		(%)	≤10 mmHg	11–15 mmHg	>15 mmHg
**Overall MAP**	1080	4.9±3.7	12.2	−2.4	0.9876	50 (4.9%)	1036(95.9%)	27(2.5%)△	17(1.6%)△
**Bloodletting**									
T0	40	5.5±2.5	10.4	0.6	0.9666	1(2.5%)	39(97.5)	1(2.5)^△^	0(0)
T1	40	10.9±6.2	23.0*	−1.2	0.9428	15(37.5)^#^	20(50.0)	9(22.5)^△^	11(27.5)^△^
T2	40	5.3±4.1	13.3*	−2.7*	0.9445	2(5)	35(87.5)	4(10)^△^	1(12.5)^△^
T3	40	3.9±2.5	8.8	−1.0	0.9455	1(2.5)	39(97.5)	1(2.5)^△^	0(0)
T4	40	3.4±2.1	7.5	−0.7	0.9524	0(0)	40(100.0)	0(0)	0(0)
T5	40	3.2±2.5	8.1	−1.7	0.9370	1(2.5)	40(100.0)	0(0)	0(0)
T6	40	2.9±2.8	8.4	−2.6	0.9379	2(5)	40(100.0)	0(0)	0(0)
T7	40	3.0±2.3	7.5	−1.5	0.9704	1(2.5)	40(100.0)	0(0)	0(0)
T8	40	3.2±2.8	8.7	−2.3	0.9673	3(7.5)^#^	40(100.0)	0(0)	0(0)
**Vasoconstriction**									
T0	40	4.2±2.6	9.3	−0.9	0.9845	0(0)	40(100.0)	0(0)	0(0)
T1	40	7.1±10.0	26.7*	−12.5*	0.8412	13(32.5)^#^	29(72.5)	6(15.0)^△^	5(12.5)^△^
T2	40	6.6±4.0	14.4*	−1.2	0.9623	3(7.5)^#^	36(90.0)	4(10.0)^△^	0(0)
T3	40	6.1±2.3	10.6	1.6	0.9866	0(0)	39(97.5)	1(2.5)	0(0)
T4	40	5.4±2.7	10.7	0.1	0.9823	1(2.5)	39(97.5)	1(2.5)	0(0)
T5	40	5.1±1.7	8.4	1.8	0.9929	0(0)	40(100.0)	0(0)	0(0)
T6	40	4.7±2.0	8.6	0.8	0.9912	0(0)	40(100.0)	0(0)	0(0)
T7	40	4.3±2.4	9.0	−0.4	0.9876	1(2.5)	40(100.0)	0(0)	0(0)
T8	40	4.2±2.5	9.1	−0.7	0.9865	1(2.5)	40(100.0)	0(0)	0(0)
**Fluid infusion**									
T0	40	4.7±2.7	10	−0.6	0.9835	1(2.5)	40(100.0)	0(0)	0(0)
T1	40	4.2±3.9	11.8	−3.4	0.9444	4(10.0)^#^	40(100.0)	0(0)	0(0)
T2	40	4.5±2.6	9.6	−0.6	0.9689	0(0)	40(100.0)	0(0)	0(0)
T3	40	4.2±2.8	9.7	−1.3	0.9711	0(0)	40(100.0)	0(0)	0(0)
T4	40	4.9±2.7	10.2	−0.4	0.9758	0(0)	40(100.0)	0(0)	0(0)
T5	40	4.9±2.3	9.4	0.4	0.9834	0(0)	40(100.0)	0(0)	0(0)
T6	40	5.2±2.2	9.5	0.9	0.9874	0(0)	40(100.0)	0(0)	0(0)
T7	40	4.9±1.8	8.4	1.4	0.9912	0(0)	40(100.0)	0(0)	0(0)
T8	40	5.6±2.1	9.7	1.5	0.9878	0(0)	40(100.0)	0(0)	0(0)

CI, Confidence Interval; Percent Error, Percent Exceeding the 95% CI of the overall difference; *r*, Correlation coefficient; Upper and Lower, the upper and the lower limit of the 95% CI; T_0_ = 0 sec of each treatment; T_1_ = 15 sec after starting each treatment; T_2_ = 30 sec after starting each treatment; T_3_ = 45 sec after starting each treatment; T_4_ = 60 sec after starting each treatment; T_5_ = 75 sec after starting each treatment; T_6_ = 90 sec after starting each treatment; T_7_ = 105 sec after starting each treatment; T_8_ = 120 sec after starting each treatment; MAP, mean arterial pressure; FA, femoral artery; CVA, caudal ventral artery. Superscript(*), disagreement according to the overall 95% CI; Superscript(^#^), number and percentage of the plots that exceed the overall 95% CI; Superscript(^△^), the frequency of individual gradients greater than those that indicate good for agreement.

The second portion of this method was the analysis of MAP measurements between the FA and the CVA at each time point under various acute hemodynamic changes (Table 3 and Figs. S2, S3). Consistent with our result that the acute hemodynamic changes have significant effects on MAP gradients (*P*<0.05), the agreement of MAP measurements between the FA and the CVA decreased during the early stage of bloodletting and vasoconstriction. The MAP gradients and the 95% limits of agreement at T_1_ during the period of bloodletting (Table 3 and Fig. S2) and vasoconstriction (Table 3 and Fig. S3) were significantly higher than the overall gradients and the limits of agreement. The mean MAP gradients at T_1_ during bloodletting (Table 3 and Fig. S2A, B) and vasoconstriction (Table 3 and Fig. S3A, B) were 10.9±6.2 mm Hg and 7.1±0.0 mm Hg, respectively. Therefore, their 95% CI were higher than 15 mm Hg (Table 3), indicating an overwhelming underestimation of MAP from the CVA under these situations. The agreement decreased at T_2_ during the period of bloodletting (Table 3 and Fig. S2C, D) and vasoconstriction (Table 3 and Fig. S3C, D) although the readings were in the range considered acceptable.

The data distribution was presented as the percentage error and the percentages of individual absolute gradients of ≤10 mm Hg, 11–15 mm Hg and >15 mm Hg in Table 3. The Concordance correlation coefficient (*r*) of each time point was also shown. Most of the gradients (95.9%) were good or acceptable, with only 1.6% of gradients exceeding the acceptable limit of 15 mm Hg. Most data that exceeded the acceptable limit or the limit of the overall gradients were concentrated in the early stage of each acute hemodynamic change.

## Discussion

In the present study, we tested a new BP monitoring site for continuous invasive blood pressure monitoring, the caudal ventral artery in the tail of both male SD rats and Wistar rats, and compared the values with the preferred monitoring site of the femoral artery. Based on the degree of agreement and safety, our main finding was that, although systemic gradients of MAP measurements between the CVA and the FA were demonstrated, both MAP values appear to be interchangeable with an overall difference of −2.5–12.3 mm Hg, regardless of whether Wistar or SD rats were hypothermic or undergoing blood-letting, fluid infusion or vasoconstrictive drug administration. None of the rats experienced tail ischemia, whereas the rate of distal ischemia after FA monitoring was quite high (25%).

Many factors should be considered when choosing an ideal BP monitoring site. These factors include the accuracy and the sensitivity of the measurement that best reflects perfusion of vital organs, the convenience to collect multi-functional, comprehensive data and diagrams, the rate and severity of complications and the retaining time associated with protection techniques. Selection of the monitoring site should ultimately be guided by the study objectives to ensure that the techniques chosen are appropriate for the experimental questions being explored and do not affect the interpretation of results [1]. Among the factors mentioned above, accuracy of measurement and the rates of related complications are the main determining factors.

In practice, direct recording of BP using fluid-filled catheters through the FA is more widely used during *in vivo* experiments in rats because it represents the aortic pressure well [1]. Thus, direct recording of the BP through the FA was regarded as the reference standard evaluation method to confirm the safety, accuracy and sensitivity of BP values simultaneously obtained from the CVA. To our knowledge, little work has been performed on invasive arterial BP monitoring using the CVA, and no work has been performed on the agreement of BP measurements between the CVA and the FA. Consistent with our research, many previous studies have shown high rates of hind limb ischemia and motor dysfunction after FA catheterization. This may mislead the investigator, particularly in neurological and behavioral studies. In contrast and to our surprise, none of the 20 rats in experiment 2 experienced distal ischemia after CVA catheterization.

To our knowledge, only a few studies have focused on the evaluation of BP in the CVA of rats compared to central arteries such as the FA or the CA [25], [26], [27]. Although the overall agreement between the CVA and the FA in previous studies seems to have drawn consistent conclusions and correspond to the results in this study, these results should be interpreted with caution. First, some of the comparative studies adopted an indirect method for reading, such as the tail cuff technique, for comparison with the direct intra-arterial readings. Though the tail cuff technique does not cause surgical injury in conscious rats, the indirect measurements provide limited information on the systolic BP. The reproducibility and variability of tail cuff recording largely depends on critically managing many factors. The environmental conditions and experimental procedures must be carefully controlled because BP can be highly influenced by HR, anesthesia, stress, and the structure or function of a specific artery. This may reduce the accuracy of tail cuff readings, causing disagreement between both of the arteries being monitored. Therefore, it can be argued that it is inappropriate to compare tail cuff readings with invasive arterial readings. Second, some validation studies have relied on simple correlation or regression analysis that can be misleading and obscure large individual differences or even systemic differences between measurement methods or monitoring sites [25]. When more appropriate analytical techniques such as agreement analysis have been used, BP measurements obtained using the tail cuff method have shown poor reliability and agreement with those simultaneously obtained by intra-arterial methods [26], [27]. Spontaneous variation arising from the intrinsic lability of BP is emerging as an important factor in cardiovascular target organ perfusion, so it is difficult and unnecessary to evaluate the absolute accuracy of the CVA pressure. On the contrary, the bias and the limits of agreement between the FA and the CVA should be carefully analyzed under similar experimental situations, similar physiological or pathophysiological states with the same technique.

MAP and HR have shown a relatively high variability in conscious, freely moving rats. The caudal ventral arterial waveforms shown in this study are clearer and more stable than those obtained in a previous study [27], in part because we obtained them under optimal anesthesia. This provided us with sensitive and comparable measurements of the SBP, DBP and MAP than previously published work. The normal range of MAP from the CVA that we obtained was 75∼120 mm Hg, which is consistent with previous results [14], [15]. Based on this result, all data were divided into two sets to facilitate a detailed analysis during experiments of the most common phenomena. One set was obtained under stable hemodynamic states; the other was obtained during acute hemodynamic changes. The overall mean difference indicated systemic gradients in SBP and MAP values. However, only the gradients of MAP values are acceptable. There were significant increase in gradients between the CVA and the reference FA measurements during the early stages of bloodletting and vasoconstriction, and this difference lasted for less than 30 sec even at low rectal temperatures or during hemorrhagic shock. It is understood that the MAP is regarded as the most appropriate index to assess the perfusion of organs. These results indicated that the CVA may be used to monitor invasive BP in healthy adult male Wistar and SD rats, but caution should be taken when unstable hemodynamic states are experienced.

The mechanisms of the development of the gradient are not completely understood. In this study, we found that all SBP values obtained from the FA were much higher than those obtained from the CVA, while DBP values obtained from the FA were similar to those obtained from the CVA. That is, the MAP gradients are much smaller than the SBP gradients. Nevertheless, the phenotypic aspects of the BP curve differ markedly when taken from the CVA or the FA, indicating that the genotype aspects and the structure of the two arteries may differ from each other. Souza et al. [4] found that the proximal segment of the CVA has a diameter compatible with a conductance vessel but not a resistance vessel. In contrast, Franca et al. [28] found that the rat tail artery develops a high perfusion pressure in response to a 2.5 ml/min flow when compared to other vascular beds. In the present study, we found that the elevation of BP is slightly earlier when measured from the CVA than from the FA. All of these phenomena indicate the heterogeneity of structure and function between these arteries.

Some limitations and methodological aspects must be considered. Though we have validated the agreement between both sites in healthy adult SD and Wistar male rats, it is not clear that whether similar results could be obtained in the female, immature or critically ill rats (e.g., sepsis or acidosis) or other strains of rats (e.g., spontaneously hypertensive rats, SHR). To remove the significant influence of local temperature changes on the arterial blood flow of the CVA, we selected the root of the tail as the puncture site, the same site as for a tail cuff. However, we did not validate other puncture sites along the proximal segment according to the results of Souza et al. [4]. Further studies should be undertaken to achieve a more comprehensive understanding of the agreement of MAP values between the CVA and the FA.

In conclusion, invasive BP monitoring of the CVA in healthy adult male SD and Wistar rats can be safely performed during experiments *in vivo*. Our findings further illustrate systematic gradients in SBP and MAP measured between the CVA and the FA sites. Based on the previous published data and the findings in this study, the maximum limits of MAP gradients of the CVA are acceptable. The CVA can be a new site for invasive arterial pressure monitoring during a relatively stable hemodynamic state regardless of the rat strain, rectal temperature or moderate changes of vascular tone and blood volume.

## Supporting Information

Figure S1 **The overall agreement analysis and the agreement analysis between the MAP values obtained from FA and CVA at T_0_ before bloodletting (baseline).** A, B, The overall agreement analysis of the total 1080 pairs of simultaneous MAP values. C, D, The agreement analysis of the 40 pairs of simultaneous MAP values at T_0_ before bloodletting. A, C, Scatter diagram and correlation coefficient (*r*) of MAP values measured at the FA and the CVA simultaneously. B, D, Brand - Altman plot of MAP values measured at the FA and the CVA simultaneously. The gradients of simultaneous MAP measurements with FA and CVA are drawn against the mean of MAP_FA_ and MAP_CVA_, ignoring the repeated nature of the data. The zero line is shown as a dotted line. The mean gradient line is shown as a solid line. The dashed lines represent the upper and the lower limits of the 95% CI of each mean gradient. They do not exceed the a priori criteria of 15 mm Hg. The small gradients and the narrow 95% limit of agreement suggest the interchangeability of both measurements.
(ZIP)

Figure S2 **The agreement analysis between the MAP values obtained from FA and CVA at T_1_ and T_2_ during bloodletting.** A, B, The agreement analysis of the 40 pairs of simultaneous MAP values at T_1_ during bloodletting. C, D, The agreement analysis of the 40 pairs of simultaneous MAP values at T_2_ during bloodletting. A, C, Scatter diagram and correlation coefficient (*r*) of MAP values measured at the FA and the CVA simultaneously. B, D, Brand - Altman plot of MAP values measured at the FA and the CVA simultaneously. The gradients of simultaneous MAP measurements with FA and CVA are drawn against the mean of MAP_FA_ and MAP_CVA_, ignoring the repeated nature of the data. The zero line is shown as a dotted line. The mean gradient line is shown as a solid line. The dashed lines represent the upper and the lower limits of the 95% CI of each mean gradient. B, The 95% limits of agreement exceed the a priori criteria of 15 mm Hg at T_1_. The gradients and the 95% limits of agreement at T_1_ do not suggest the interchangeability of both measurements. D, The 95% limits of agreement at T_2_ exceed the overall 95% CI. This indicated that the concordance of MAP values measured at the FA and the CVA decreased at T_2_.
(ZIP)

Figure S3 **The agreement analysis between the MAP values obtained from FA and CVA at T_1_ and T_2_ during vasoconstriction.** A, B, The agreement analysis of the 40 pairs of simultaneous MAP values at T_1_ during vasoconstriction. C, D, The agreement analysis of the 40 pairs of simultaneous MAP values at T_2_ during vasoconstriction. A, C, Scatter diagram and correlation coefficient (*r*) of MAP values measured at the FA and the CVA simultaneously. B, D, Brand - Altman plot of MAP values measured at the FA and the CVA simultaneously. The gradients of simultaneous MAP measurements with FA and CVA are drawn against the mean of MAP_FA_ and MAP_CVA_, ignoring the repeated nature of the data. The zero line is shown as a dotted line. The mean gradient line is shown as a solid line. The dashed lines represent the upper and the lower limits of the 95% CI of each mean gradient. B, The 95% limits of agreement exceed the a priori criteria of 15 mm Hg at T_1_. The gradients and the 95% limits of agreement at T_1_ do not suggest the interchangeability of both measurements. D, The 95% limits of agreement at T_2_ exceed the overall 95% CI. This indicated that the concordance of MAP values measured at the FA and the CVA decreased at T_2_.
(ZIP)
