# Correction: Comparison of Invasive Blood Pressure Measurements from the Caudal Ventral Artery and the Femoral Artery in Male Adult SD and Wistar Rats

**DOI:** 10.1371/annotation/c997cd3e-68ea-4e5f-82ca-6443c618b439

**Published:** 2013-09-10

**Authors:** Ying Wang, Yushuang Cong, Jun Li, Xueting Li, Bing Li, Sihua Qi

The fifth author, Bing Li, should be noted as a Corresponding Author, and her email address is icecreamlee@hotmail.com. 

