# Supplementary figures and images for: Comparison of Invasive Blood Pressure Measurements from the Caudal Ventral Artery and the Femoral Artery in Male Adult SD and Wistar Rats
Source: PLoS One. 2013 Apr 5;8(4):e60625. doi: 10.1371/journal.pone.0060625 (PMC3618036; doi:10.1371/journal.pone.0060625)

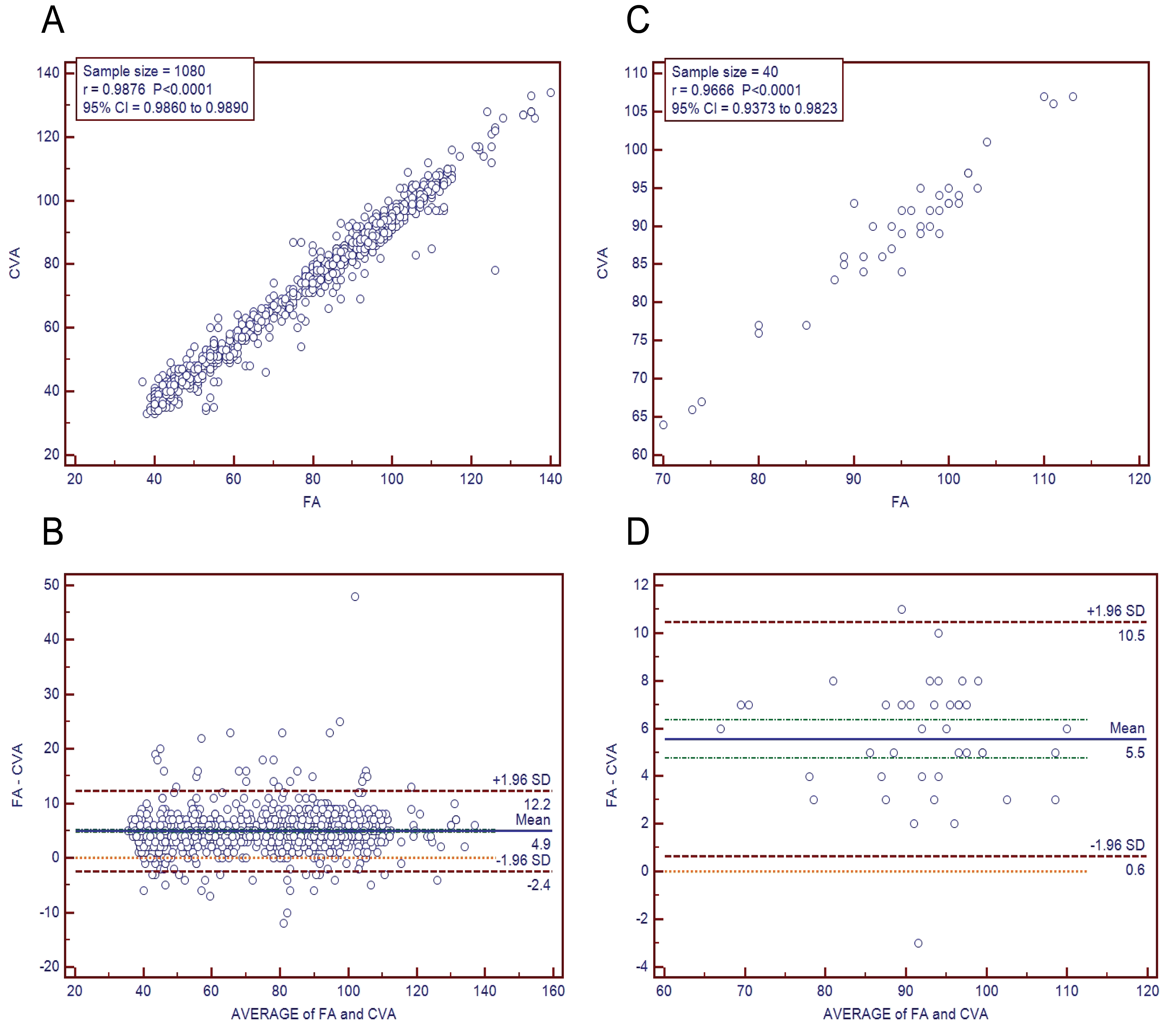

Supplement: Figure S1 — The overall agreement analysis and the agreement analysis between the MAP values obtained from FA and CVA at T0 before bloodletting (baseline). A, B, The overall agreement analysis of the total 1080 pairs of simultaneous MAP values. C, D, The agreement analysis of the 40 pairs of simultaneous MAP values at T0 before bloodletting. A, C, Scatter diagram and correlation coefficient (r) of MAP values measured at the FA and the CVA simultaneously. B, D, Brand - Altman plot of MAP values measured at the FA and the CVA simultaneously. The gradients of simultaneous MAP measurements with FA and CVA are drawn against the mean of MAPFA and MAPCVA, ignoring the repeated nature of the data. The zero line is shown as a dotted line. The mean gradient line is shown as a solid line. The dashed lines represent the upper and the lower limits of the 95% CI of each mean gradient. They do not exceed the a priori criteria of 15 mm Hg. The small gradients and the narrow 95% limit of agreement suggest the interchangeability of both measurements. (ZIP) [file pone.0060625.s001.zip › PONE-D-13-00921-Figure S1.tif.tif]

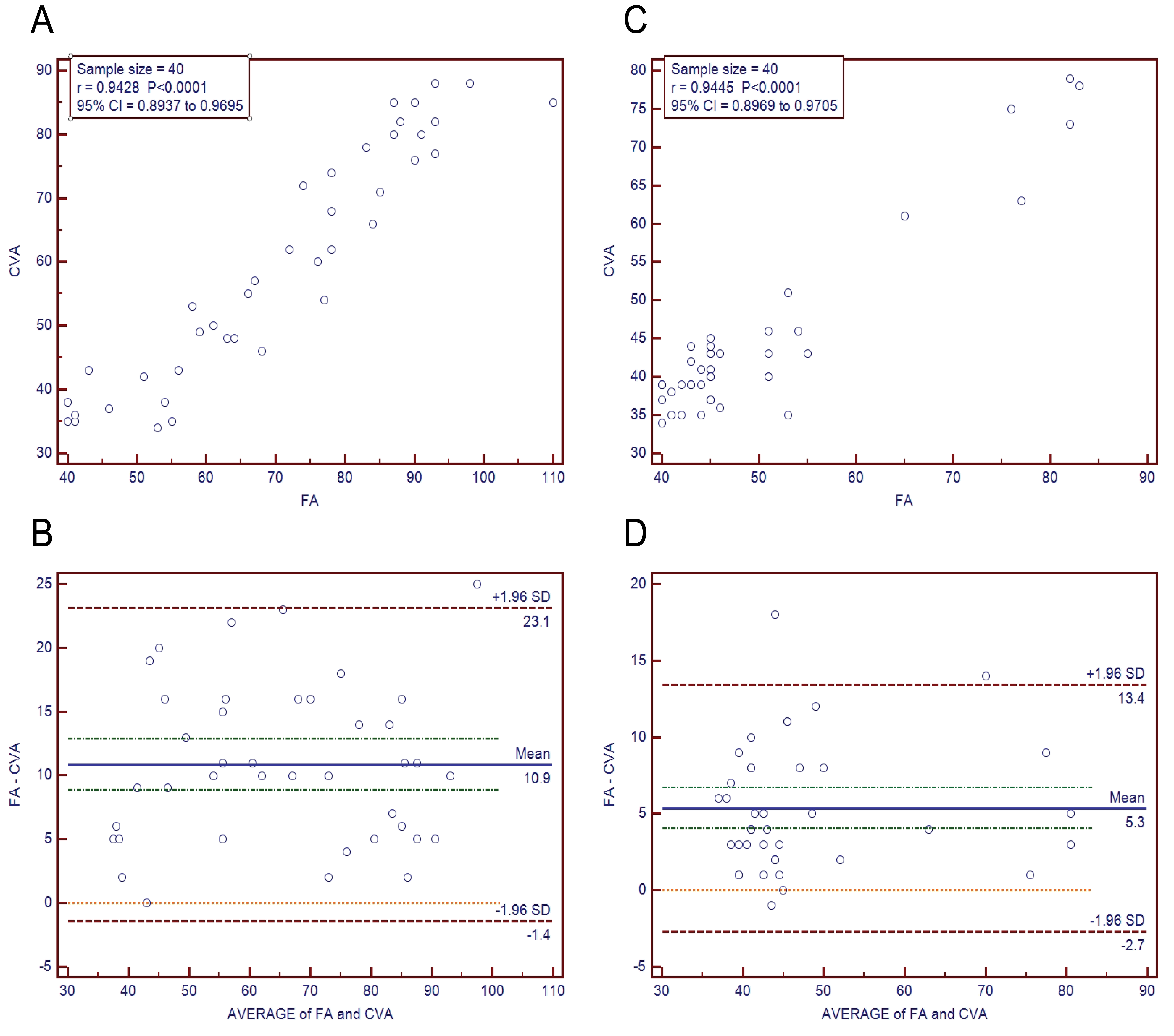

Supplement: Figure S2 — The agreement analysis between the MAP values obtained from FA and CVA at T1 and T2 during bloodletting. A, B, The agreement analysis of the 40 pairs of simultaneous MAP values at T1 during bloodletting. C, D, The agreement analysis of the 40 pairs of simultaneous MAP values at T2 during bloodletting. A, C, Scatter diagram and correlation coefficient (r) of MAP values measured at the FA and the CVA simultaneously. B, D, Brand - Altman plot of MAP values measured at the FA and the CVA simultaneously. The gradients of simultaneous MAP measurements with FA and CVA are drawn against the mean of MAPFA and MAPCVA, ignoring the repeated nature of the data. The zero line is shown as a dotted line. The mean gradient line is shown as a solid line. The dashed lines represent the upper and the lower limits of the 95% CI of each mean gradient. B, The 95% limits of agreement exceed the a priori criteria of 15 mm Hg at T1. The gradients and the 95% limits of agreement at T1 do not suggest the interchangeability of both measurements. D, The 95% limits of agreement at T2 exceed the overall 95% CI. This indicated that the concordance of MAP values measured at the FA and the CVA decreased at T2. (ZIP) [file pone.0060625.s002.zip › PONE-D-13-00921-Figure S2.tif.tif]

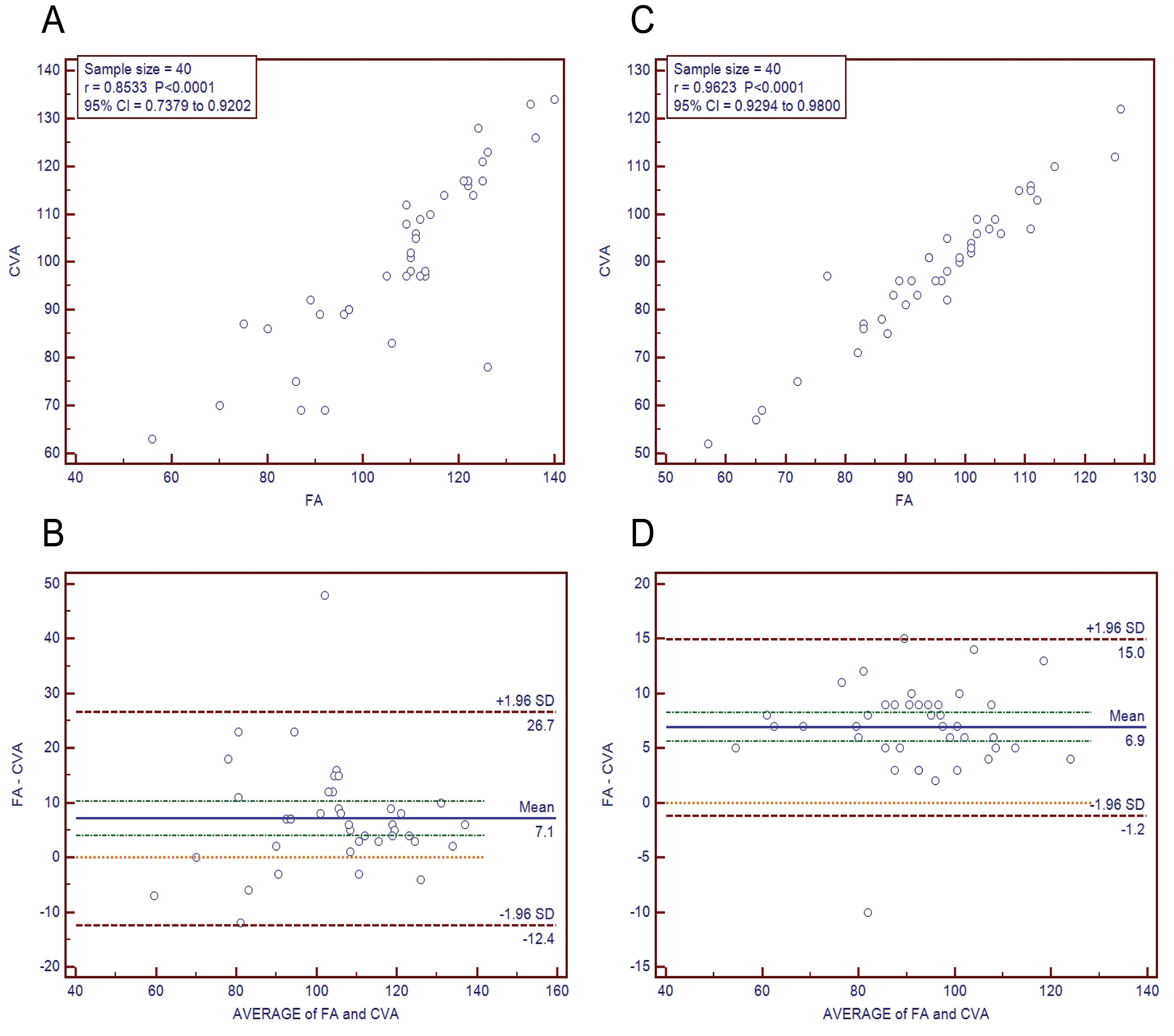

Supplement: Figure S3 — The agreement analysis between the MAP values obtained from FA and CVA at T1 and T2 during vasoconstriction. A, B, The agreement analysis of the 40 pairs of simultaneous MAP values at T1 during vasoconstriction. C, D, The agreement analysis of the 40 pairs of simultaneous MAP values at T2 during vasoconstriction. A, C, Scatter diagram and correlation coefficient (r) of MAP values measured at the FA and the CVA simultaneously. B, D, Brand - Altman plot of MAP values measured at the FA and the CVA simultaneously. The gradients of simultaneous MAP measurements with FA and CVA are drawn against the mean of MAPFA and MAPCVA, ignoring the repeated nature of the data. The zero line is shown as a dotted line. The mean gradient line is shown as a solid line. The dashed lines represent the upper and the lower limits of the 95% CI of each mean gradient. B, The 95% limits of agreement exceed the a priori criteria of 15 mm Hg at T1. The gradients and the 95% limits of agreement at T1 do not suggest the interchangeability of both measurements. D, The 95% limits of agreement at T2 exceed the overall 95% CI. This indicated that the concordance of MAP values measured at the FA and the CVA decreased at T2. (ZIP) [file pone.0060625.s003.zip › PONE-D-13-00921-Figure S3.tif.tif]
